# Forefront Users’ Experience Evaluation by Employing Together Virtual Reality and Electroencephalography: A Case Study on Cognitive Effects of Scents

**DOI:** 10.3390/brainsci11020256

**Published:** 2021-02-18

**Authors:** Marco Mancini, Patrizia Cherubino, Giulia Cartocci, Ana Martinez, Gianluca Borghini, Elena Guastamacchia, Gianluca di Flumeri, Dario Rossi, Enrica Modica, Stefano Menicocci, Viviana Lupo, Arianna Trettel, Fabio Babiloni

**Affiliations:** 1 BrainSigns Srl, Via Lungotevere Michelangelo, 9, 00192 Rome, Italy; patrizia.cherubino@brainsigns.com (P.C.); giulia.cartocci@brainsigns.com (G.C.); ana.martinez@brainsigns.com (A.M.); gianluca.borghini@brainsigns.com (G.B.); elena.guasta@gmail.com (E.G.); gianluca.diflumeri@brainsigns.com (G.d.F.); stefano.menicocci@brainsigns.com (S.M.); viviana.lupo@brainsigns.com (V.L.); arianna.trettel@brainsigns.com (A.T.); fabio.babiloni@brainsigns.com (F.B.); 2 Department of Economics, Management and Business Law, University of Bari Aldo Moro (UniBa), Via Camillo Rosalba, 53, 70124 Bari, Italy; 3 Department of Molecular Medicine, Sapienza University of Rome, Viale Regina Elena, 291, 00161 Rome, Italy; 4 Department of Communication and Social Research, Sapienza University of Rome, Via Salaria, 113, 00198 Rome, Italy; 5 IRCCS Fondazione Santa Lucia, Neuroelectrical Imaging and BCI Lab, Via Ardeatina 306, 00179 Rome, Italy; 6 Department of Anatomical, Histological, Forensic & Orthopedic Sciences, Sapienza University of Rome, Piazzale Aldo Moro, 5, 00185 Rome, Italy; dario.rossi@uniroma1.it (D.R.); enrica.modica@uniroma1.it (E.M.)

**Keywords:** Electroencephalography (EEG), virtual reality (VR), mental effort, neuroscience, scent, smell, lavender, lemon

## Abstract

Scents have the ability to affect peoples’ mental states and task performance with to different extents. It has been widely demonstrated that the lemon scent, included in most all-purpose cleaners, elicits stimulation and activation, while the lavender scent elicits relaxation and sedative effects. The present study aimed at investigating and fostering a novel approach to evaluate users’ experience with respect to scents’ effects through the joint employment of Virtual Reality and users’ neurophysiological monitoring, in particular Electroencephalography. In particular, this study, involving 42 participants, aimed to compare the effects of lemon and lavender scents on the deployment of cognitive resources during a daily life experience consisting in a train journey carried out in virtual reality. Our findings showed a significant higher request of cognitive resources during the processing of an informative message for subjects exposed to the lavender scent with respect to the lemon exposure. No differences were found between lemon and lavender conditions on the self-reported items of pleasantness and involvement; as this study demonstrated, the employment of the lavender scent preserves the quality of the customer experience to the same extent as the more widely used lemon scent.

## 1. Introduction

### 1.1. Virtual Reality: A Realistic Experience Preserving a High Ecological Validity

Nowadays, we are witnessing a huge growth of Virtual Reality (VR) and its related applications as well as of the employment of VR in research contexts. The technological advances performed by the gaming industry allow researchers to design highly immersive virtual environments (HIVE) by means of game engines, such as Unity3D [1] and Unreal Engine [2], where the “nature” of the virtual environments is represented by the motion in a simulated virtual geographic space, often bound to game missions or to the experience of a “spatial story”. Game engines have become widely used to develop HIVE which, differently from common cartography criteria [3], allow for the representation of geospatial data in VR with a high detail level generating the vivid illusion of being in a virtually mediated environment, a phenomena that has been called *spatial presence* [4]. VR has already been shown to be a powerful solution in several research fields, from military training to clinical applications for treating mental disorders as phobias or post-traumatic stress disorder (PTSD) [5,6,7].

One of the main drivers of VR is the *sense of presence* defined by the sensation experienced by users, who are exposed to the computer-generating environment, of being there [8,9], along with the degree of *immersion* that is related to the technological system [10]. In particular, the main components that enhance the *immersion sensation* refer to graphical realism, multimodal integration, interactivity [11], sounds, and emotional atmosphere [12]. In this direction, an univocal agreement emerged by previous studies: the sensation of being present increases according with the level of immersion offered by VR technology [12,13,14,15] and users are more likely to perceive a higher immersion and sense of presence when the interaction with the environment is mediated by sophisticated technology such as Head Mounted Display (HMD) compared to less sophisticated technologies [5,13,16].

The growing number of HMD manufacturing companies is causing a drop in the HMD prices [17,18] as well as a simultaneous increase in the availability and differentiation of HMD models and the launch of new products that are capable to generate the illusion of real vision. The enhanced illusion relies on the lenses located in the device that are inspired to the eye’s functioning and to how the brain processes the visual inputs and is capable to eliciting complex mental processes [5]. 

The investigation of human responses in the daily life experience, both from a cognitive and emotional point of view, is more complicated than expected because of the limitation in reproducing an ecological environment in a laboratory setting and in controlling the wide variety of variables that get involved in the experience. Indeed, research protocols based on the presentation of media and aimed to the investigation of emotional and cognitive processes often fail to recreate a realistic and ecological settings generating a loss of validity and the consequent difficulties in properly and reliably exploring mental states [9,12]. VR could play a key role in this frame as it allows researchers to reproduce ad-hoc immersive 3D environments where users’ perceptions can reach high levels of realism, and any object can be manipulated in real time. Furthermore, researchers have already noticed the potentiality of VR, compared to traditional tools, for studying emotional reactions and mental states [19,20], and in the manipulation of the environment. This latter aspect is crucial in the modulation of our experiences as already demonstrated by traditional paradigms such as the stimulus-organism-response (S-O-R) [21]. 

Therefore, VR tools and techniques represent an opportunity for researchers to work with highly controlled environments where many aspects of the experience can be easily manipulated in a controlled way. It is important to mention that such manipulation can be referred not only to VR objects, such as scenes and environments, but it can also involve variables coming from the real world and capable to affect the perception of the individuals while interacting with a highly controlled VR environment. In particular, the investigation of smell is extremely interesting for researchers when it is performed in VR because even if a scent is manipulated outside of the VR world, it can still affect human behavior within the VR world while people observe a stimulus or interact with an object.

### 1.2. The Power of Scents

Olfaction represents one of the most ancient sense from the evolutionarily point of view and one of the most complex sense to investigate considering also that it takes place in many life functions [22]. Scent engages several structures of the human brain such as the piriform cortex, located in the ventral frontal-temporal junction and playing an important role in the identification of the smell, and the thalamus that distributes the smell information to other structures like the orbitofrontal cortex, the hippocampus, and the amygdala [23,24]. The capability of the scents to trigger memories and emotions is strictly associated to the latter mentioned brain regions that are historically known to be involved in memorization and emotional processing [25,26] and that are directly linked to the olfactory receptors located in the human nose [27,28]. In contexts in which the emotional response elicited by an odor and the odor identification play a key role, some authors demonstrated that the close link between the emotional quality of an odor and its identification relies on the two affective dimensions of valence and dominance [29].

A recent study on scent perception demonstrated an increment of theta oscillation at the level of the piriform cortex while olfaction stimuli were processed. Such an increment mediated the olfactory processing by coordinating and exchanging the information between the pyriform cortex and hippocampus. Moreover, this study proved that odor elicits theta power selectively in the human piriform cortex within 500 ms of a sniff, and odor-specific content can be decoded from piriform oscillations as early as 110 ms [30]. Other studies described the role of the alpha frequency band in the perception of olfactory stimuli [31,32]. Di Flumeri et al. [32] showed how pleasant and unpleasant scents produce different patterns in the alpha activity. Furthermore, the self-reported perceptions were aligned with the contribution provided by the EEG activity: when the subjects evaluated a scent as pleasant, an asymmetric alpha activity over the prefrontal cortex was observed. The same event was observed and demonstrated while subjects appreciated a picture or other kind of sensorial stimuli [33,34,35]. Other studies showed how the activity in different EEG frequency bands can be affected by the use of specific fragrances. For example, the presence of jasmine flavor is associated with an increase of alpha activity [36], while the inhalation of a mixture of lavender and bergamot is associated with an increase of theta activity in the right prefrontal lobe [37]. 

Valuable insights have been provided by previous research focused on the smell investigation and its effect on human experience in several fields. For instance, within the marketing field, fragrances judged to be pleasant and congruent have been found to be capable to affect the product evaluation [38,39], the time spent in a store [40,41,42], and the purchase behavior [41]. Furthermore, it is important to highlight that not every environmental cue is consciously perceived as stimuli coming from the external world are also processed beyond the threshold of human awareness. In support of this assumption, some empirical evidence have proved that scent may modulate human perception unconsciously [43]. 

### 1.3. Neurophysiological Evaluation of User’s Experience

Different neuroimaging techniques have been proposed to catch insights about users’ mental states during a particular experience reproduced in laboratory or even in close-to-real settings [44,45,46,47,48,49,50,51]. The most used technologies are the functional Near-InfraRed spectroscopy (fNIRs) and the Electroencephalography (EEG). However, the latter is definitely the most used one because of its great compromise between technology cost, portability, usability, and performance in terms of spatial and time resolution [46]. The recent technological advancements and the development of wearable solutions and dry sensors are undoubtedly establishing EEG as the main research tool for human behavior studies [52,53]. 

In this context, EEG has been employed to investigate video gamers’ experience [54,55]. For example, Naumann and colleagues proposed an EEG-based system that was able to predict the difficulty level of a video game [56]. In particular, EEG signals were recorded by using a 32-electrodes device from 6 participants playing a modified Tetris game under 10 different difficulty levels. The proposed approach was able to predict the levels with a high accuracy yielding a mean prediction error of less than one level. EEG is now often used also in marketing research. The so-called *consumer neuroscience* investigates the possibility to access information within the consumer’s brain during the generation of a preference or the observation of commercial advertisements [57,58,59,60,61,62] by using EEG-based neurometrics. In the last years, several studies have been published showing how it is possible to detect hidden signs of memorization process and emotional engagement like pleasantness [63,64,65,66]. Such indexes have been successfully applied also for the evaluation of auditory [65,67], olfactory [32,68], and tasting [35,69] stimuli. 

### 1.4. The Case Study

In this scenario, the present work aimed at investigating the feasibility and capability in using VR and EEG to investigate users’ experiences with respect to the particular scents vaporized. 

Considering the contribution provided by previous research, this study aimed to shed more light on the effect of relaxing/activating scent on mental effort, during an ordinary experience like a train journey, employing lavender and lemon scents that are respectively associated with relaxation [31,70,71], and activation [72]. An important aspect to consider is that through virtual reality was possible for the participants to face two train journeys with ad-hoc features (e.g., specific scents diffusion), where all the events of the experience, of interest or not, have been presented at specific time in each experimental session (the flow of events over time was always the same for all subjects). The impossibility of controlling the variables of interest (scents diffusion) and environmental variables (e.g., noise, people interaction and other distractors), together with the obvious economic reasons related to the rent of two real trains from two different railway companies to face a trip between Rome and Milan for research purposes, makes the idea of running a similar research in a real train highly unattainable. For those reasons, virtual reality was used as a holistic approach [73], and allowed us to present stimuli taking into account the “veridical control of laboratory measures and the verisimilitude of naturalistic observation of real life situations” [74,75] and reach an optimal balance between the need for exacting control over key variables and the naturalistic observation [76]. 

This study focused on exploring the impact that such fragrances could have on the participants’ mental effort deployed to process two key messages: the generic message (welcome voice), and the informative message (delay voice). In addition, it is important to mention that the level of mental effort was monitored to detect also potential differences between scent conditions (i.e., lavender vs. lemon) over the entire train journey. Along with the lavender, the choice to employ the lemon fragrance rather than other fragrances like rosemary or jasmine was made taking into account the ordinary olfactive experience of the individuals during a train journey. Besides being associated with the idea of cleaning, lemon aroma is typically used as aroma for all-purpose cleaners [40,70,77,78] and can be considered a congruent odor with the tested environment (i.e. virtual train). In this context, the opposite effect of lavender and lemon can represent a novelty aspect if related to the ability to deploy cognitive resources allocated to process stimuli that play a crucial role in the customer experience and that are administered through holistic approach [73] with high ecological validity.

According to our intentions, the EEG has been used to quantify the mental effort elicited by the stimuli of interest that is defined as the “amount of cognitive resources employed by a subject while engaged in a task”. Scientific literature related to the mental effort and workload widely demonstrated that theta activity over the prefrontal brain cortex significantly increases whit high levels of mental effort, and vice versa [79,80]. Therefore, EEG frontal theta activity is now considered as a reliable indicator of mental effort of subjects involved in different tasks, such as car driving [81,82], air traffic control [83,84,85], mathematical tasks [86,87], perception of complex auditory stimuli [88], and task performance in general [89,90]. Beyond the neurophysiological investigation performed through the computation of the Global Field Power (GFP) in the theta band over the frontal channels (index of mental effort), self-reported measures of pleasantness, involvement, audio perception, and smell awareness were considered in order to provide further cues related to the general perception of the train journey in condition of exposure to lemon or lavender.

The main experimental questions of the present study were:

Is it possible to employ VR together with EEG to evaluate user’s experience, and in particular the effect of specific scents on user’s cognitive capabilities in understanding informative audio messages?

Is it possible to detect significant differences in terms of mental activity, in particular mental effort, while exposed to two different scents, i.e., lavender and lemon, supposed to induce two different moods, respectively, relaxation and activation?

## 2. Materials and Methods

### 2.1. Participants

The experimental sample was composed of 42 healthy participants (21 females and 21 males; mean age = 28.6 years; SD = 6.7 years). The participants, recruited on a voluntary basis, self-reported to have no history of mental illnesses or drug abuse. The study was conducted in accordance with the principles outlined in the Declaration of Helsinki of 1975, as revised in 2008, and approved by the Sapienza University of Rome Ethical Committee (Ethic approval code: 191126_BRAIN) in charge for the Department of Molecular Medicine. After the explanation of the study, each participant signed a document of informed consent which included the authorization to use the video-graphical material (i.e., photos and videos of the experiment).

### 2.2. Experimental Groups

The whole experimental group was divided into two experimental subgroups (21 participants each): one was exposed to the lemon scent, while the other was exposed to the lavender one. In order to avoid any sickness or similar psychophysiological disorder due to a prolonged experience in VR [91], the experiment duration was limited as much as possible, therefore it was not possible to ask the participant to repeat the experience in both the conditions. At the beginning of the experimental session, each participant was randomly assigned to the “lemon” group and the “lavender” group using a computer-based randomization procedure (“Research Randomizer” tool, available at the following link: https://www.randomizer.org (accessed on 17 February 2021)), based on the random assignment by blocks method in order to preserve an equal sample size for both groups.

During the experiment, each group was indeed exposed to the either the lemon scent or the lavender scent. The scent was diffused in the air of the experimental room by the use of the same vaporizing diffuser device with a continuous flow, constant intensity, and handling the same amount of essential oil (i.e., spray capability equal to 350 ml of liquid per hour). When the participant entered the experimental room, the lavender or lemon scent was already present in the air and would have been present for the entire “VR train journey” experience (see Section 2.3.3).

### 2.3. Experimental Protocol

**Figure 1:**
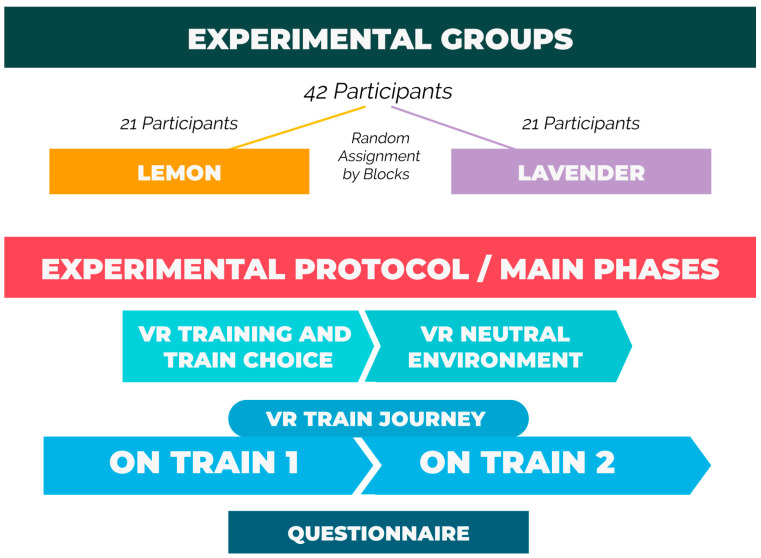
Distribution of participants across the two conditions “lemon” and “lavender”, and the four main phases of the experimental protocol: “VR training and train choice”, “VR neutral environment (baseline)”, “VR train journey” (on train 1 and train 2), and “Questionnaire”.

The experimental protocol consisted in four main phases (see Figure 1) named as follows: “VR training and train choice” (5 minutes of total duration), “VR neutral environment—baseline” (1 minute of total duration), “VR train journey” (11 minutes of total duration, 5:30 minutes on each train) and “Questionnaire” (around 3 minutes of total duration). Participants’ brain activity (i.e., EEG signal) was recorded along the entire experimental protocol except for the “VR training and virtual train choice” and “Questionnaire” phases. The entire experience in virtual reality has been kept under the 20 minutes to avoid the intromission of potential VR sickness symptoms [92], fatigue, and sensory adaptation that would negatively affect the goodness of the research. Each main phase is explained in detail in the following sections.

#### 2.3.1. VR Training and Train Choice

**Figure 2:**
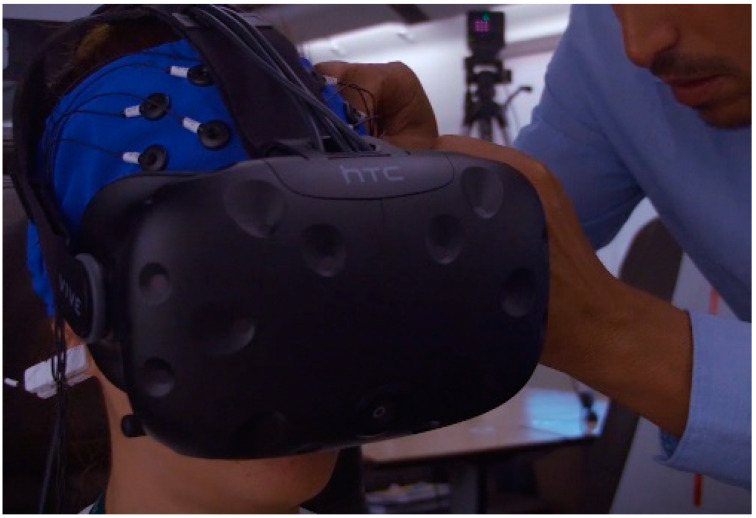
Electroencephalography (EEG) and Virtual Reality (VR) equipment setup.

After the EEG setup, the participants were invited to wear a head mounted display (HMD, HTC Vive model) and headphones (see Figure 2) to take part in a short training experience allowing them to familiarize with a general virtual reality environment. The VR training experience lasted around 3 minutes and was carried out in separated room where no scent was vaporized. During this phase, the participants were immersed in a virtual apartment and were asked to walk around and turning their heads in order to be aware of the 360-degree tracking, movement, and VR audio properties. 

In this context, the participants were introduced to the tasks that they would have to performed during the following phases. In particular, within the same VR training environment, they were shown a timetable related to several high-speed trains departing from Rome to Milan and were asked to choose two trains they would have liked to travel by, among those included in the timetable.

The only requirement related to this choice was to not choose two trains from the same company. All the trains available on the timetable were related to one of the two Italian leading companies for high-speed rail transport, “Frecciarossa” and “Italo”. In fact, the two train companies are different in terms of interior design: in this way we ensured that there was not any bias due to other external variables since all the participants experienced both the trains.

Furthermore, participants were warned that some of the high-speed trains could experience delays during the trip because of a technical issue on the high-speed rail line and that any delay would affect the length of the virtual trip as well as the duration of the whole experiment. This latter statement was addressed in order to stimulate a true reaction in participants, in term of mental processes, to the delay communication presented in each virtual train. In order to further preserve this aspect, we did not informed participants beforehand on the duration of the two trips scenario and we did not tell them if their chosen trains would experience a delay or not.

#### 2.3.2. VR Neutral Environment (Baseline)

After the train choice, the subjects were invited to relax while immersed in a simple virtual environment for 1 minute. This environment was represented by a white room with no textures and objects, designed with the aim of recreating a basic/neutral environment to be used as baseline for the EEG data processing.

#### 2.3.3. VR Train Journey 

During the next phase, each participant, still wearing the HMD and the EEG headband, was invited to the experimental room where the lemon scent or the lavender scent had been already diffused in the air according to the specific subject inclusion in one of the two distinct groups (i.e., lemon or lavender).

For each subject, the protocol consisted of two virtual high-speed train trips, one by “Frecciarossa” and the other by “Italo”. The order of exposure to both trains was randomized using the random order assignment method of the “Research Randomizer” tool (available at the following link: https://www.randomizer.org (accessed on 17 February 2021)), in order to avoid any effect related to the presentation order.

**Figure 3:**
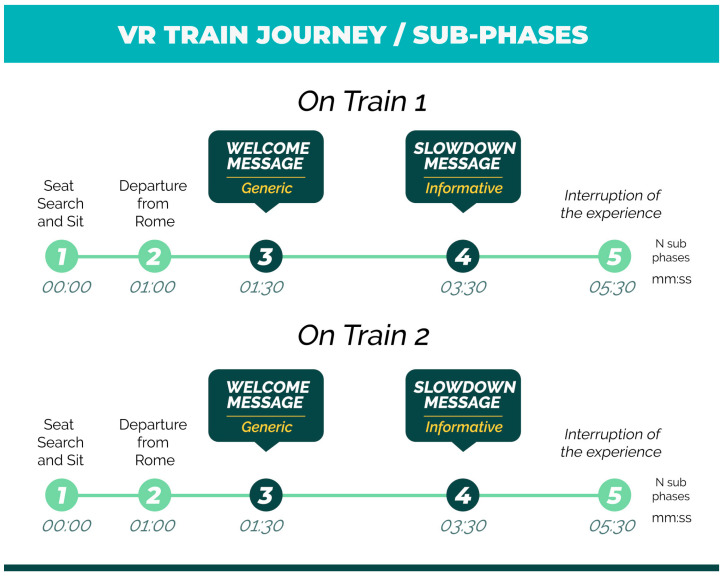
VR train journey: each subphase has been rename with the name of the most representative event happening in that specific time interval. The most significant events, for the purposes of the current study, were represented by the “welcome message” (generic message) and the “slowdown message” (informative message).

Each virtual trip consisted in the same sub-phases (see Figure 3) and the entire virtual experience, considering both trains, lasted around 11 minutes.

Sub-phase 1—Seat search and sit: the participant was immersed in a virtual reality environment consisting in the interior of a high-speed train (“Frecciarossa”/“Italo”) and was invited to find the assigned seat and then to sit down. A real chair, of the same dimensions of the virtual seat, was located according with the position of the virtual seat.

**Figure 4:**
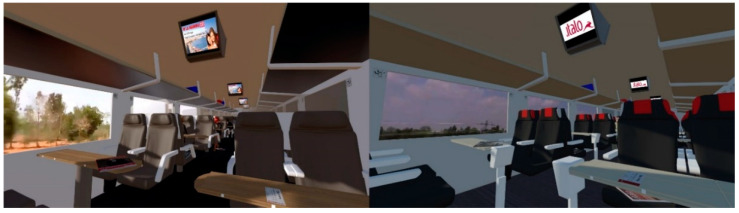
Interior of the “Frecciarossa” (left side) and “Italo” (right side) high-speed virtual trains.

Sub-phase 2—Departure from Rome: One minute after the beginning of the experience, the train started moving. The effect associated with the movement of the train was achieved by employing a video of a flowing external landscape visible through the train windows (see Figure 4) along with the presence of several sound effects designed to increase the perception of realism.

Sub-phase 3—Welcome message (Generic): 30 s after the train’s departure, the “on board voice” gave the welcome to the travelers. The welcome message had a total duration of 15 s. The entire scene associated to this mentioned sub-phase can be watched in the first half of the video available at the link https://youtu.be/xxUsKSiQy-Y (accessed on 17 February 2021).

**Figure 5:**
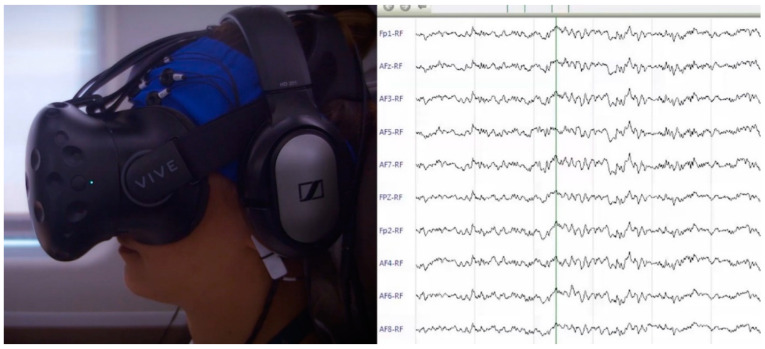
Brain activity (i.e., EEG signal) recording of one participant during virtual high-speed train trip.

Sub-phase 4—Slowdown message (Informative): At the minute 3:30 from the beginning of the experience, the “on board voice” warned the travelers that the train was undergoing a slowdown because of a technical issue on the high-speed rail line, apologizing for the inconvenience. The slowdown message had a total duration of 10 s (Figure 5). The entire scene associated to this mentioned sub-phase can be watched in the second half of the video available at the link https://youtu.be/xxUsKSiQy-Y (accessed on 17 February 2021).

Sub-phase 5—Interruption of the experience: At the time stamp 5:30, the participant virtual experience suddenly terminated leaving him/her for some seconds immersed in a dark virtual environment with no audio sources. The researchers explained to the subject that this interruption was due to a sudden malfunction of the HMD and that, for such reason, the virtual experience on the first train had to be quit. 

We introduced such false statement because we needed to keep the entire virtual reality experience as short as possible to avoid the intromission of potential VR sickness symptoms that would affect the goodness of the research [92]. Furthermore, we decide the use a false statement that relied on external causes, not-subject related and quite common to experience in virtual reality applications to avoid generating stress in participants and preserve a realistic reaction to the delay also for the second train trip. If we disclosed that the duration of each train trip was equal to 5:30 minutes, such a disclosure would have undermined the credibility of the second virtual trip as well as a realistic reaction to the delay.

Afterwards, the researchers recommended to the participant to be ready again, because the trip on the second high-speed train would start in the next few seconds. The same procedure was repeated for the second high-speed train trip. 

#### 2.3.4. Questionnaire

Once each participant’s entire virtual experience on both the trains ended, the HMD and the EEG frontal headband were removed to allow the participant to comfortably sit down in another room and fill in a short questionnaire. During this phase, the participant was asked to rate the items *pleasantness*, *involvement*, *audio-perception* (this latter further divided in “audio involvement”, “audio identification” and “audio localization”) relatively to the entire virtual experience ranging from 1 (minimum) to 7 (maximum). In addition, subjects were invited to report if they were aware of the presence of a distinct smell within the experimental room, and in case of awareness to report the name of the perceived fragrance.

#### 2.3.5. Data Recording and Signal Processing

**Figure 6:**
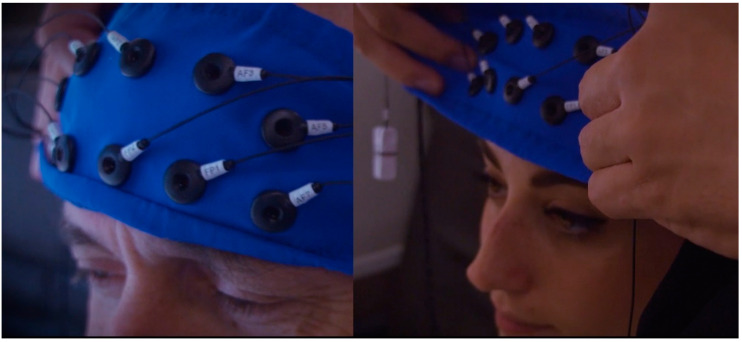
EEG frontal headband with ten electrodes (Fpz, Fp1, Fp2, AFz, AF3, AF4, AF5, AF6, AF7, and AF8).

A ten electrode (Fpz, Fp1, Fp2, AFz, AF3, AF4, AF5, AF6, AF7, and AF8) EEG frontal headband along with a portable 21-channel system (BEmicro and Galileo software, EBneuro, Italy) was utilized to record the EEG activity with sampling rate of 256 Hz (Figure 6). The EEG electrodes were grounded at the left earlobe and referred to the right earlobe. All the skin-electrode impedances were kept below 10 kΩ.

The recorded data were processed offline through MATLAB software (MathWorks Inc., Natick, MA, USA). EEG signals were digitally bandpass filtered by a 5th order Butterworth filter ([2 ÷ 30] Hz) to remove the continuous component and high-frequency interferences, and then a Notch filter (50 Hz) was applied.

Afterwards, the Independent Component Analysis (ICA) was performed in order to remove artefactual components. In particular, the SOBI algorithm was employed [93]. The signal was decomposed into 10 Independent Components (ICs, equal to the number of EEG channels), two specific ICs related to eye blinks and saccades artefacts have been visually identified by an expert, and then the EEG signal was reconstructed [94] and re-referenced by means of the common average reference (CAR). 

However, this conservative approach does not ensure a completely artefact-free EEG data. In order to remove any other kind of artefacts that is not univocally associated to one IC, further automatic procedures of the EEGLAB toolbox [95] were adopted in order to remove corrupted portions of data. The EEG signal was segmented into 1-second-long epochs, shifted of 0.5 s in order to avoid any “boundary effect”, and then the following epoch rejection criteria have been applied to automatically recognize artefact data epochs [96]:

(i) Threshold criterion: EEG epochs with a signal amplitude exceeding ±100 μV were labeled as artefact.

(ii) Trend criterion: EEG epochs were interpolated in order to check the slope of the trend within the considered epochs. If the slope of an epoch was higher than 10 μV/s, the considered epoch was marked as “artefact”. 

(iii) Sample-to-sample difference criterion: The EEG epoch was marked as “artefact” also if the amplitude difference between consecutive EEG samples was higher than 25 μV, inasmuch it would represent a no-physiological variation.

Accordingly to the epoch rejection criteria [96], which use has been validated in different previous studies [84,97,98], EEG epochs labelled as “artefact” were removed from the EEG dataset, in order to obtain an EEG dataset free of artefacts. The 12 ± 3% of data has been on average removed.

At the beginning of the EEG recording, the participants were invited to close their eyes for 60 s. Such time interval was then employed for the calculation of the individual alpha frequency (IAF), i.e., the peak of the signal power spectrum within the traditional alpha frequency range (8–12 Hz). According to Klimesch [99], the IAF was then used to define the individual EEG frequency bands of each participant as follows: theta [IAF − 6–IAF − 2], alpha [IAF − 2–IAF + 2], beta [IAF + 2–IAF + 16] and gamma [IAF + 16–IAF + 30]. Therefore, the EEG signal of all the electrodes was filtered in the individual theta frequency band, being the most informative band related to mental effort variations as described in (see Section 1.4).

Afterwards the global field power (GFP) was obtained, in order to get a summary of the activity related to the cortical areas of interest in a specific frequency band [100], in this case theta activity over the frontal electrodes. 

Several studies focusing on attentional and cognitive processing [101,102] as well as clinical studies [103,104] performed the calculation of the GFP that can be considered as a reference-independent descriptor of the field potential [105]. 

Below is the GFP formula in the form of an equation that allowed us to calculate the average GFP value on all the GFP values estimated over 1 second of EEG signal:

(1) $$GFP_{\vartheta ,Frontal}=\;\frac{1}{N}\overset{N}{\underset{i=1}{\sum }}x_{\vartheta_{i}}^{}{\left(t\right)}^{2}$$

where *ϑ* represents the specific EEG band, Frontal represents cortical area, *N* represents the number of electrodes related to a specific area, *i* represents the electrodes’ index and *x* is the specific EEG sample at time *t*, filtered within the related EEG band (i.e., *ϑ*) and for the specific channel *i*. 

The EEG-based mental effort neurometric, useful to evaluate the participants’ mental effort [88,106], was calculated as the GFP in Theta band over all the frontal electrodes (Fp2, AF4, AF6, AF8, AF7, AF3, Fp1, AF5). In fact, an increasing frontal theta activity was demonstrated to be correlated to increasing mental effort [79,82], would result in an increasing of the aforementioned *GFPFrontal,ϑ* index, thus this can be finally employed as the neurometric of the participants’ mental effort. 

Finally, the neurometric of mental effort was normalized for each second using the mean and the standard deviation of the same neurometric calculated on the individual baseline (see “VR neutral environment” in Figure 1):

(2) $$normalized\;index=\frac{index-mean_{baseline}}{standard\;deviation_{baseline}}$$

#### 2.3.6. Performed Analysis

Independent samples t-tests were performed considering the “scent” as grouping variable. This variable had two levels, “lemon” and “lavender”, based on the experimental groups’ definition (see Figure 1).

The test variables were instead represented by the mental effort neurometric related to three main time segments of interest, representative of the experiences on both high-speed virtual trains: 

“Whole experience” (5:30 minutes of total duration): this variable referred to the time segment attributable to one entire high-speed train trip. Such variable was computed from the average of the mental effort neurometric values obtained for the two high-speed train trips in association to the mentioned time segment of interest.

“Generic message” (15 s of total duration): this variable referred to the time segment in which the “generic message” (welcome message) was provided by the on-board voice. This variable was computed from the average of the mental effort neurometric values obtained for the two high-speed train trips in association to the mentioned time segment of interest.

“Informative message” (10 s of total duration): this variable referred to the time segment in which the “informative message” (slowdown message) was provided by the on-board voice. Such variable was computed from the average of the mental effort neurometric values obtained for the two high-speed train trips in association to the mentioned time segment of interest.

For each subject, the Mental Effort Neurometric was averaged between the two trains experience, in a coherent way for each segment of interest, to avoid any particular bias due to train interiors, as explained in Section 2.3.1.

Regarding the questionnaire variables associated to the self-reported measures, independent samples t-test was performed considering such variables as test variables, while the “scent” was considered as a grouping variable with two levels, “lemon” and “lavender”, based on the experimental groups’ definition (see Figure 1). In order to increase data readability and cross comparisons, the mentioned variables were normalized between 0 and 1. 

Concerning the self-reported measure of *awareness*, related to the presence of a distinct smell, no statistical analysis could be performed. However, such data will be reported to provide a better frame for the discussion of results. 

## 3. Results

### 3.1. Mental Effort

**Figure 7:**
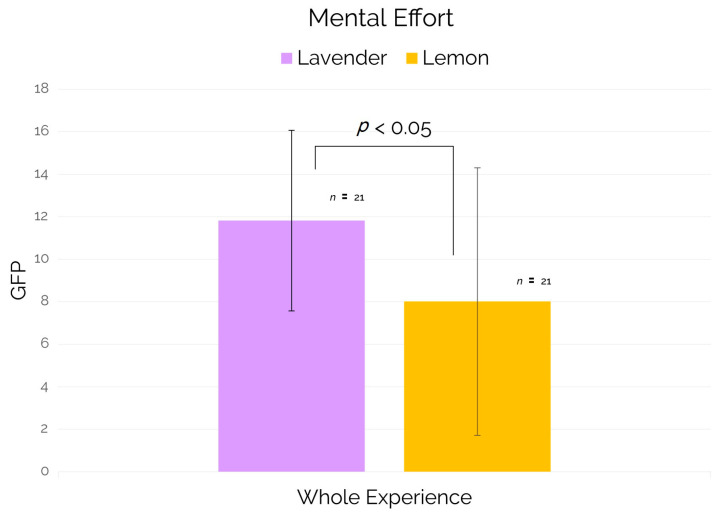
Mental Effort for the "whole experience" time segment.

Regarding the mental effort neurometric (ME) averaged for both trains and related to the entire experience (“whole experience”), the results showed a statistically significant increase (*p* = 0.028) for ME values in subjects exposed to the lavender scent in comparison to those ones exposed to the lemon scent (see Figure 7).

**Figure 8:**
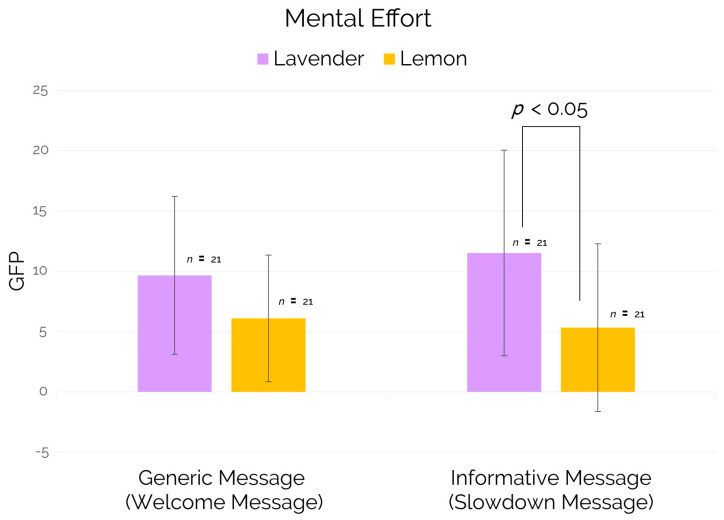
Mental Effort for the time segments associated respectively to the generic message (welcome message) and the informative message (slowdown message).

Regarding the mental effort neurometric (ME) averaged for both trains and related to the generic message (welcome message) and to the informative message (slowdown message), the results showed a statistically significant increase (*p* = 0.023) for ME values in subjects exposed to the lavender scent in comparison to those ones exposed to the lemon scent, during the occurrence of informative message (see Figure 8). A similar trend was found also for the generic message (welcome message) but in this case the comparison of the two experimental groups (lemon and lavender) on the mental effort neurometric showed no significant differences.

### 3.2. Self-Reported Smell Awareness and Smell Identification by Name

**Figure 9:**
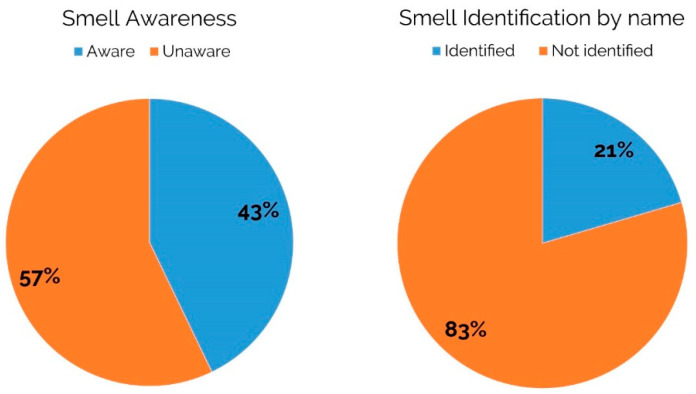
Self-reported measure of “smell awareness” and “smell identification by name”, provided by subjects at the end of the experimental session.

Despite the fact that all subjects were exposed to a specific smell (lemon/lavender) during the virtual reality experience, more than half of the sample (57%) stated that they were unaware of the presence of a smell (see Figure 9).

Furthermore, only the 21% of the participants correctly recognized by name the specific fragrance to which they were exposed (lavender/lemon).

### 3.3. Self-Reported Pleasantness and Involvement

**Figure 10:**
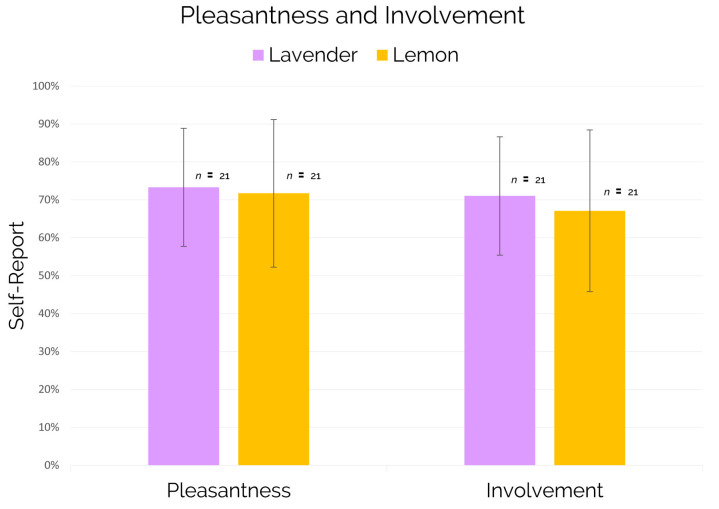
Self-reported measures of pleasantness and involvement associated to the entire virtual reality experience and provided by subjects at the end of the experimental session.

Regarding the self-reported measures of pleasantness and involvement, the results showed no statistically significant differences for subjects exposed to the lavender scent in comparison to those ones exposed to the lemon scent (see Figure 10).

### 3.4. Self-Reported Audio Perception (Involvement, Identification, Localization)

**Figure 11:**
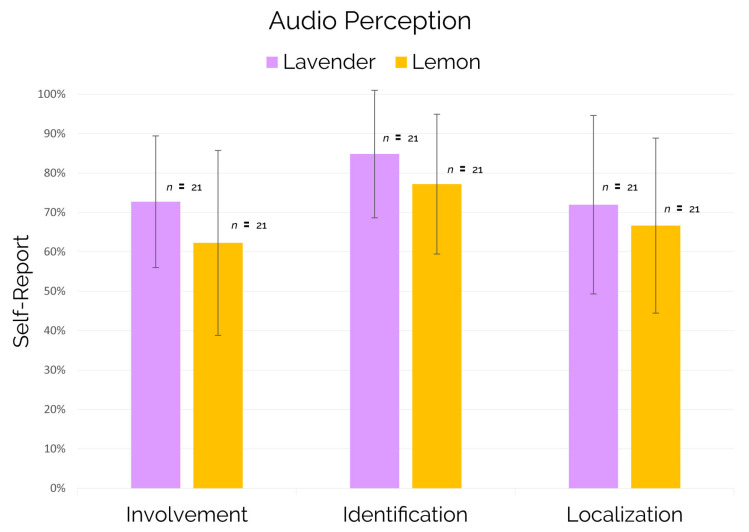
Self-reported measures of "audio involvement", "audio Identification", "audio localization" associated to the entire virtual reality experience and provided by subjects at the end of the experimental session.

Concerning the self-reported measures “audio involvement”, “audio identification”, and “audio localization” associated to the audio perception of the entire experience on both virtual high-speed trains, the results showed no statistically significant differences between subjects exposed to the lavender scent and subjects exposed to the lemon scent (see Figure 11).

## 4. Discussion

### 4.1. Summary

The present study explored the impact of lavender scent in comparison to the lemon one in terms of mental effort demand when processing audio messages, especially during a VR-simulated train journey.

Lavender is typically considered a sedative-type aroma whose inhalation has been associated to a decrease in the level of autonomic nervous system (ANS) arousal (e.g. decrease of blood pressure, heart rate, and skin temperature) [107] and to the active performance of tasks in terms of quality, accuracy and reaction time [108,109,110,111]. In addition, previous research showed that lavender was capable to improve concentration and work efficiency [112]. On the other hand the lemon aroma has been traditionally associated to activation [72] and employed in all-purpose cleaners, like train cleaning [40,70,77,78], representing a congruent odor with the tested environment (virtual train). 

The above mentioned and opposite features associated to lavender and lemon led us to narrow the comparison to those specific fragrances and focusing our attention on the deployment of cognitive resources that has been investigating in the EEG frontal theta activity, known as a reliable indicator of mental effort [81,82,83,84,85,86,87,89,90].

Our results showed a significant increment of mental effort for subjects exposed to the lavender scent in comparison to subjects exposed to the lemon scent during the entire virtual travel experience (see Figure 7). 

Considering the relaxing properties and the sedative effect of the lavender scent showed by previous research [72,110], the results provided by the present work seems to indicate that a calm state creates the condition for a cognitive disclosure and the predisposition for a better elaboration of the information provided by the environment. Furthermore, the association between lavender and improvement in cognitive functions is consistent with previous studies [110,111].

Regarding the deployment of cognitive resources allocated for processing the audio messages, the results showed a significant higher mental effort in subjects exposed to the lavender scent than subjects exposed to the lemon scent only during the occurrence of the informative message (see Figure 8). As mentioned in previous sections, considering that the informative message consisted in the on-board voice warning related to the presence of an inconvenience (e.g. slowdown due to a technical issue on the high-speed rail line), and that each subject, at the beginning of the experimental session, was advised that a similar inconvenient would have increased the duration of the virtual trip as well as of the experiment, this message category had a direct impact on the participant experience differently from the generic message (*welcome voice*). In other words, the results of the present study would suggest that the lavender scent seems therefore to be more powerful than the lemon for a better cognitive processing and comprehension of relevant and informative content. Of course, these results should be confirmed by a large-scale study including more “stimuli” conditions and a control group. However, these results reveal that EEG-based neurometrics can be sensitive to cognitive phenomena dynamics due to scents exposure even in a VR environment.

Furthermore, the present work showed no differences in terms of self-reported involvement and pleasantness associated to the entire virtual trip between the lavender and lemon condition (see Figure 10) assuring that the use of the lavender scent, over lemon, would not negatively affect the customer experience.

Regarding the self-reported measures “audio involvement”, “audio identification” and “audio localization” associated to the audio perception of the entire experience on both virtual high-speed trains, even if the trend illustrated in Figure 11 could be indicative again of a better contribution of the lavender over lemon, such differences did not reach the significance. 

Undoubtedly, the present study would have benefit from testing both the scents with all the participants, even including a “scent-free” control condition, however while dealing with VR a critical constraint of the experimental design is the risk of inducing sickness and other psychophysiological disorders to the participants [91]. This phenomenon is highly subject-dependent and hard to monitor and predict, therefore the experiments must be designed limited to the purposes of the study. This concern could be even more serious in this kind of neuroscientific studies since some cues of sickness could affect human physiological activities. Therefore, in the present study the VR experience duration was kept as short as possible. 

The obtained results provided intriguing perspectives regarding the joint use of VR and neurophysiological monitoring, in particular EEG, for the evaluation of users’ experiences. Even though it is a preliminary study with some intrinsic limitations, which are discussed in detail here below, the proposed setup allowed us to point out significant effects in mental effort allocated to understand an audio message while exposed to different scents. The proposed setup becomes particularly of interest when the research target is not the users’ overt behavior but the even unconscious mental states and reactions. Not surprisingly, in terms of participants’ awareness related to the perception of a scent, our data showed that more than half of the sample (57%) was unaware of the presence of an ambient scent (Figure 9). 

### 4.2. Recommendations for Future Experimental Studies

Some limitations of the study and recommendations for future studies should also be discussed and will be reported below:

• First, even if virtual reality allows the generation of a high controlled environment that allows to preserve a high ecological validity, caution is required when integrating such technology with neuroscientific tools, like the EEG. In particular, before running the experiment with real subjects, we performed several trials in order to identify potential issues related to such integration. We found out that some brands of EEG were affected by electrical interferences more than others due to the proximity of the electrodes and the HMD streaming the VR images. Moreover, during such trials we noted that the use of the frontal headbands with dry sensors returned a significant amount of noise due to the contact of the headband with the HMD and to the constant movement of participants. For these reasons we used an EEG device that was not affected by such electrical interference, standard EEG gel-based electrodes, and we also limited subjects’ movement asking them to remain seated for most of the virtual reality experience. 

• Second, in order to further investigate the differences in the cognitive perception of the informative and generic message in condition of lavender/lemon scent, the integration of further self-reported measures specifically related to those messages would be recommended. In our study we added several items with the aim to explore the accuracy of the audio perception, but they were related to the entire experience which also included environmental noises. In this direction, the results obtained and illustrated in Figure 11 showed an interesting trend, again in favor of the lavender scent, but they did not reach the significance. We believe that the administration of similar items, but specific for the generic and the informative messages, could provide further insights and direct link between self-reported and neurophysiological measures.

• Third, we did not apply any method aimed to identify the subjective detection-threshold related to the perception of the odors. Our attention was mainly focused in replicating a daily life situation and in keeping constant the spray amount, the speed, and intensity related to the diffusion of the scent for the two tested odors. Nevertheless, the application of methods that allows to identify the subjective detection-threshold related to the perception of the odors, could also provide further variables to consider for a deeper exploration of the self-reported measures of “smell awareness” (see Figure 9).

• Four, there is an ongoing debate in the field of spatial cognition whether constant speed motion has different effects on spatial performance measures rather than teleport motion (which has been established as reliable and user-friendly approach in video and computer games to move in VR space). Further studies could therefore investigate if and how different motion techniques would affect the cognitive processing of scents in virtual reality.

Considering the above limitations and recommendations we believe that future studies will be able to provide further insights about the effect of the scent on mental states within an experimental frame where VR meets neuroscience. In particular, the combination of technologies such as the EEG and HMD in the same experimental protocol requires cautio, because of the high risk of noise contamination. Moreover, the integration of methods aimed to identify the subjective detection-threshold related to the perception of the odors along with a collection of self-reported data specifically oriented to the informative and generic messages, could be at the base of future valuable findings.

## 5. Conclusions

In a world where the lemon scent is typically used as an aroma for all-purpose cleaners, like those ones usually employed for the train cleaning, the results provided by this study indicate the lavender scent as a powerful alternative capable to elicit a greater amount of cognitive resources during the environment processing than the lemon scent.

In general, the relaxing effect operated by the lavender fragrance seems to encourage a higher deployment of cognitive resources with a much greater extent than the lemon, traditionally associated to stimulation, activation and alert.

In a context where the amount of empirical works conducted on lemon scent is far below than those ones aimed to investigate the lavender effect and considering that a previous study showed that lemon, like lavender, seems to have positive effects on task performance while dealing both with mental e physical tasks [72], our study sheds more light on the effective use of lavender scent, compared to lemon scent, in terms of the deployment of cognitive resources when processing audio messages. Furthermore, it is important to highlight that the use of lavender over lemon does not negatively affect the perception of involvement and pleasantness associated to the customer experience (see Figure 10) and its employment represents a valid solution in order to positively affect the task performance and concentration levels confirming and extending findings reported by previous research [112].

Regarding the processing of generic and informative communications, this study provided interesting evidence that could be taken into account by marketing experts in order to optimize their strategies in several fields. The use of lavender over lemon, was indeed indicative of a significant higher deployment of cognitive resources allocated for the processing of informative communication represented by information relevant to the purpose of the individual (see Figure 8). In this direction, if many beneficial effects of the scent have been already provided by previous research focusing on product evaluation and purchase behaviors [38,39], our study demonstrated how well the integration of neuroscientific and virtual reality tools could represent an added value to the marketing field of the customer experience to explore psychological phenomena associated to scents within an immersive and realistic experience that preserves a high ecological validity.

Beyond marketing implications, new types of human interfaces could be developed, by exploiting the multisensory stimulation. Future wearable technology could be able to adapt the environment to human needs according to their safety. An accurate processing of informative messages in some context could even save lives (e.g., airplane ditching evacuation procedures). Wearable-Neuroscientific technology, thanks to the analysis of the neurophysiological signals, could detect the amount of cognitive resources deployed during the processing of informative messages and automatically adjust the environment (e.g. spraying lavender scent) in order to encourage the deployment of an optimal level of cognitive resources necessary for a correct processing of the message. 

In conclusion, the opportunity to study the scent effect in environments that are not even existing at the moment but that will turn into reality in the next future, preserving a high ecological validity, along with the neurophysiological investigation, represents a source of innovation and inspiration for researchers that aim to achieve valuable insights.

## Data Availability

The data presented in this study are available on request from Prof. Babiloni (fabio.babiloni@brainsigns.com).
